# Rare Case of Rapidly Worsening REM Sleep Induced Bradycardia

**DOI:** 10.1155/2015/546712

**Published:** 2015-08-16

**Authors:** Ayyappa S. Duba, Suneetha Jasty, Ankit Mahajan, Vijay Kodadhala, Raza Khan, Prithviraj Rai, Mohammad Ghazvini

**Affiliations:** Howard University Hospital, Washington, DC, USA

## Abstract

Sinoatrial arrest also known as sinus pause occurs when sinoatrial node of the heart transiently ceases to generate the electrical impulse necessary for the myocardium to contract. It may last from 2.0 seconds to several minutes. Etiologies of sinoatrial arrest can be complex and heterogeneous. During rapid eye movement (REM) sleep, sinus arrests unrelated to apnea or hypopnea are very rare and only a few cases have been reported. Here we report a case of 36-year-old male with no significant past medical history who presented to our hospital after a syncopal episode at night. Physical examination showed no cardiac or neurological abnormalities and initial EKG and neuroimaging were normal. Overnight telemonitor recorded several episodes of bradyarrhythmia with sinus arrest that progressively lengthened over time. Sleep study was done which confirmed that sinus arrests occurred more during REM sleep and are unrelated to apnea or hypopnea. Electrophysiology studies showed sinus nodal dysfunction with no junctional escape, subsequently a dual chamber pacemaker placed for rapidly worsening case of REM sleep induced bradycardia.

## 1. Introduction

Rapid eye movement (REM) related bradyarrhythmia syndrome and sinus arrest in the absence of an underlying cardiac or physiologic sleep disorder were first described in the early 1980s [1]. Very few cases have been reported suggesting that the prevalence of this nighttime rhythm abnormality is very rare. According to a literature review published in 2011, only 8 cases (mean age 34_11 years; 88% male) of REM sleep related cardiac arrest have been identified [2]. Interestingly, most of them had vague “daytime” chest pain, dizziness, and syncope as part of their symptom complex. Our patient had no daytime symptoms and could easily have been missed, if not for the only episode of syncope he had at night after he woke up from sleep.

## 2. Case Report

A 36-year-old Caucasian male with no significant past medical history presented after an episode of loss of consciousness and fall. Patient reported waking up from sleep and feeling dizzy while walking to the restroom. When he was about to urinate he passed out, fell, and hit his head. He regained consciousness as soon as he hit the floor. He denied palpitations, chest pain, or diaphoresis before or after loss of consciousness. He is a social drinker and nonsmoker and denied using any recreational drugs.

**Figure 1:**
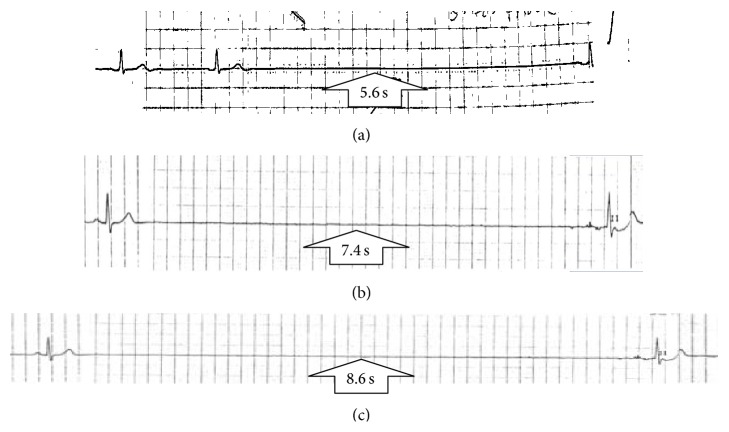
Telemetry strips during sleep showed progressively prolonging periods of sinus arrest.

On physical examination, he was found to be fully alert and had normal orthostatic vitals. Except for a 5 × 0.5 cm bruise over the right eyebrow and tenderness over the site, he had no abnormal findings. Urine toxicology screen was negative. EKG showed normal sinus rhythm at 70 beats per minute. Computed tomography of the head ruled out any acute intracranial pathology. Telemonitoring at night showed periods of sinus arrest (with the longest pause being 5.6 seconds, Figure 1(a)) with baseline bradycardia at a rate of 30 to 45 beats per minute. Patient was asymptomatic and sleeping during these pauses. Serum electrolytes, thyroid stimulating hormone, liver function tests, and serological tests for Lyme's disease were normal. Transthoracic echocardiogram showed normal ejection fraction and no valvular abnormalities. His sinus pauses progressively increased in duration (Figure 1(b)) every successive night and the longest pause lasted for 8.6 sec (Figure 1(c)). During the day, his telemetry was normal at a rate ranging between 65 and 75 beats per minute without any episodes of sinus pause. On exercise, patient showed an appropriate increase in heart rate. After a thorough literature review, a very rare diagnosis of REM sleep related bradyarrhythmia syndrome with malignant vagotonia was considered.

Polysomnographic study (Table 1) was done to evaluate sleep apnea and time sinus pauses with sleep cycle. Study did not involve video monitoring but snoring was generally absent. A few mild central respiratory events were noted. However, these did not reach frequency threshold for diagnosing sleep apnea. Cardiac telemetry showed normal sinus rhythm. During REM sleep, he demonstrated prolonged sinus pauses. Five sinus pauses of at least 3.5 seconds were noted during REM sleep. The longest one lasted for 7.8 seconds. Minimum pulse rate in wake and NREM cycle was 48.0 and 46.0, respectively. The patient appeared clinically asymptomatic all through the study and denied any symptoms compatible with REM sleep behavior disorder.

**Figure 2:**
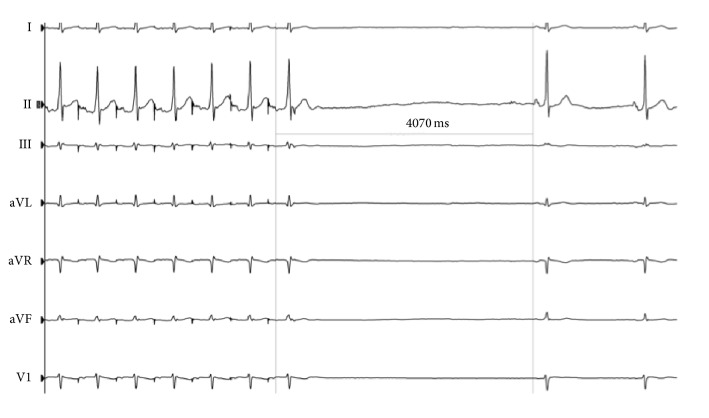
Electrophysiology study showed sinus of 4070 milliseconds (4.07 secs).

Electrophysiological study was done to further evaluate his bradycardia and need for a permanent pacemaker. Patient was found to have sinus pauses lasting up to 4070 msec (Figure 2). Considering his worsening duration of sinus pauses and lack of rescue from an escape rhythm, his risk of cardiac arrest appeared very high and a dual chamber pacemaker was placed. Pacemaker is the only established therapeutic option for these types of sinus arrests. Patient continues to follow with us as an outpatient and no episodes of syncope or night time symptoms have been reported thus far.

## 3. Discussion

REM sleep related bradyarrhythmia syndrome is an unusual REM parasomnia that is characterized by transient sinus arrest lasting several seconds during REM sleep in otherwise healthy individuals [3]. The autonomic nervous system (ANS) is a key modulator of heart rate. During sleep, various adaptations in the ANS occur. Bradycardia due to increased vagal tone and hypotension, caused by reduction of sympathetic activity, may occur during nonrapid eye movement sleep (NREM). Conversely, sympathetic activity and thus heart rate increase during rapid eye movement (REM) sleep. REM sleep related bradyarrhythmia syndrome likely reflects exaggerated vagal tone or acute withdrawal of sympathetic activity during phasic REM events. Whether this reflects central influences on the autonomic nervous system or an exaggerated baroreflex response is unknown [2].

Presentation of this nocturnal disorder is often intriguing. Among the few clinical cases reported, surprisingly all the patients who were symptomatic had diurnal symptoms. Symptoms varied among atypical chest pain, blurring of vision, fainting, lightheadedness, and more often a combination of these during the daytime. Few patients were accidentally detected while undergoing a polysomnographic study for other reasons. In REM related bradyarrhythmia with sinus arrests, since episodes occur during REM sleep, patients might remain asymptomatic. Some cases of sudden unexpected death during sleep in apparently healthy individuals can be explained by these prolonged REM sleep related sinus arrests. Unlike the previously reported cases, our patient had progressive worsening of maximal sinus pauses every successive night, which posed an imminent threat of sudden cardiac death. Only one case was previously reported in literature, where patient had a similar worsening of maximal sinus pauses [4]. However, unlike our patient, his worsening of pauses occurred at a far slower pace (over a year).

Management of these cases is often very challenging. Pacemaker implantation is the only successful therapeutic approach described in the literature for patients with symptomatic REM sleep related bradyarrhythmias and sinus arrests. In a literature review, eight patients with REM related sinus arrest were collectively analyzed [2]. Among them, 7 patients were symptomatic (diurnal symptoms) and one patient was asymptomatic. Six of the 7 symptomatic patients were eventually treated with implantable cardiac pacemakers. Of these, five patients were followed on the long-term. Interestingly, all stayed asymptomatic for a mean follow-up duration of 3.7 ± 2.9 years. Although symptomatic benefits of cardiac pacing are quite evident from this review, systematic verification of this assumption and postulating a mortality benefit is difficult due to paucity of this condition. In the absence of diurnal symptoms, we do not know whether certain cases of sudden cardiac death might have been the first presentation of this nocturnal arrhythmia [1]. Whether REM related sinus arrest predisposes to sudden nocturnal cardiac death is unknown, but implanting a pacemaker may alleviate this potential risk [2].

## 4. Conclusion

This is a unique presentation of REM sleep related bradyarrhythmia with rapidly worsening maximal sinus arrest duration. All the patients previously reported with this disorder presented with vague diurnal symptoms or were asymptomatic. Our patient presented with exclusive nocturnal symptoms (dizziness during sleep interruptions even while lying and an episode of syncope), which are more congruent with the timing of this rhythm disturbance. This will be the first reported case of REM sleep related bradyarrhythmia with exclusive nocturnal symptoms and rapidly worsening maximal sinus arrest duration.

## Figures and Tables

**Table 1:** Basic results of polysomnographic recordings.

BMI (kg/m^2^)	23.4
Total sleep time (TST)	405 minutes
REM	19.3%
Central apnea/hypopnea events	2/4
Obstructive apnea/hypopnea events	0/0
Apnea/hypopnea index	1.1
Number of AV blocks (>2.4 seconds)	0
Number of sinus arrests (>2.4 seconds)	16
Duration of cardiac pauses	2.5–7.8 secs
Sleep stage during cardiac pause	REM
Mean oxygen saturation in REM	97.9%
Minimal oxygen saturation in REM	94.5%
Minimal oxygen saturation	91.4%
% TST oxygen saturation <90%	0%
